# International case series of metastasis to penis

**DOI:** 10.1002/bco2.282

**Published:** 2023-08-30

**Authors:** Irini Youssef, Laura Elst, Nick Watkin, Hielke Martijn de Vries, Oscar Brouwer, Chris Protzel, Benjamin Ayres, Maarten Albersen, Philippe E. Spiess, Peter A. S. Johnstone

**Affiliations:** ^1^ SUNY Downstate Medical Center Brooklyn New York USA; ^2^ Moffitt Cancer Center Tampa Florida USA; ^3^ University Hospitals Leuven Leuven Belgium; ^4^ Department of Urology St George's University Hospitals NHS London UK; ^5^ The Netherlands Cancer Institute Antoni van Leeuwenhoek Hospital Amsterdam Netherlands; ^6^ Department of Urology Helios Hospital Schwerin Schwerin Germany

**Keywords:** metastatic cancer, overall survival, penis, priapism, systemic therapy

## Abstract

**Objectives:**

To evaluate clinical characteristics associated with survival in patients with metastases to the penis.

**Methods:**

After approval by the IRB, records of collaborating centres in Leuven, London, Rostock, Amsterdam and Tampa were screened for men presenting with metastatic disease to penis. Multivariate logistic regression analyses were used to identify covariables associated with survival. We analysed clinical data on 34 patients.

**Results:**

Primary sites were most frequently prostate (*n* = 14, 41%) and bladder (*n* = 9, 26%). Twenty‐eight of 34 (82%) presented with metachronous penile metastases, and 11 (32%) patients had penile metastases as the sole metastatic site. Penile metastatic locations were most frequently in the corpora (*n* = 18; 53%). Seven (21%) patients with penile metastases had priapism on presentation. Systemic therapy was frequent and variable (chemotherapy *n* = 12; immunotherapy *n* = 5; hormones *n* = 3). Local management included either surgery (*n* = 10) or RT (*n* = 8). Twelve‐ and 24‐month overall survival rate were 67% and 35%, respectively. No clinical parameter including primary histology, synchronous or metachronous metastases or priapism showed statistical survival benefit or detriment.

**Conclusion:**

Metastasis to penis arises most frequently from pelvic primaries. Priapism does not appear to correlate with survival in this large, well‐defined series.

## Abstract 2

Penile metastases are rare, with about 400 reported via case reports or small series. Under such circumstances coherent data about their behaviour may not be possible given vagaries of publication. For instance, one published meta‐analysis has proposed that malignant priapism contributes to worse survival, but this may be due to selection bias in published reports. We sought to evaluate clinical characteristics associated with survival in an international cohort of patients with metastases to the penis treated in major genitourinary cancer programmes.

## 1 INTRODUCTION

The finding of metastatic cancer to penis is rare; the literature is replete with case reports and only a single modern meta‐analysis.^1^ While this reference does relate unique information, it unfortunately is subject to multiple issues inherent in analysis of published reports: lack of comprehensive reporting, absent data and publication biases.

The centres noted here have collaborated on recent studies regarding human papillomavirus (HPV) infection, nodal radiotherapy^2^ and perineal urethrostomy^3^ for penile cancer. We sought to leverage their extensive experience in the management of secondary metastatic disease of the penis.

## 2 PATIENTS AND METHODS

After approval by the appropriate Institutional Review Boards, records of the collaborating centres were screened for men presenting with metastatic disease to penis. Lesions that resulted from direct extension from any cancer were excluded. Parameters of specific interest included primary site, synchronous versus metachronous presentation, other sites of metastasis, metastatic site within penis, existence of priapism, treatment of penile metastasis, local control duration and survival after penile metastasis. The Kaplan–Meier method was used to generate an overall survival curve. A multivariable Cox proportional hazard model was used to calculate hazard ratios (HRs) based on a panel of covariates determined a priori as mentioned above. All tests were two‐sided, and *p* < 0.05 was considered statistically significant. All analyses were performed using SPSS software system 2021, Version 28.0 (IBM, Armonk, NY).

## 3 RESULTS

Data are included in Table 1. Thirty‐four patients were documented with penile metastases since 1998. Primary sites were most frequently prostate (*n* = 14, 41%) and bladder (*n* = 9, 26%). Twenty‐eight of 34 (82%) presented with metachronous penile metastases, and 11 (32%) patients had penile metastases as the sole metastatic site. Penile metastatic locations were most frequently in the corpora (*n* = 18; 53%). Seven (21%) patients with penile metastases had priapism on presentation.

**TABLE 1:** Presenting parameters of collected patients.

Parameter	*n* (%)
Contributing centre	
Amsterdam	2 (6%)
Leuven	12 (35%)
London	9 (26%)
Rostock	2 (6%)
Tampa	9 (26%)
Primary site	
Prostate	14 (41%)
Bladder	9 (26%)
Kidney/ureter	4 (12%)
Rectal	2 (6%)
Lung	2 (6%)
Other	3 (9%)
Metastatic site	
Base	2 (6%)
Corpora	18 (53%)
Glans	8 (24%)
Overlapping/not recorded	6 (18%)
Timing	
Synchronous	6 (18%)
Metachronous	28 (82%)

**FIGURE 1:**
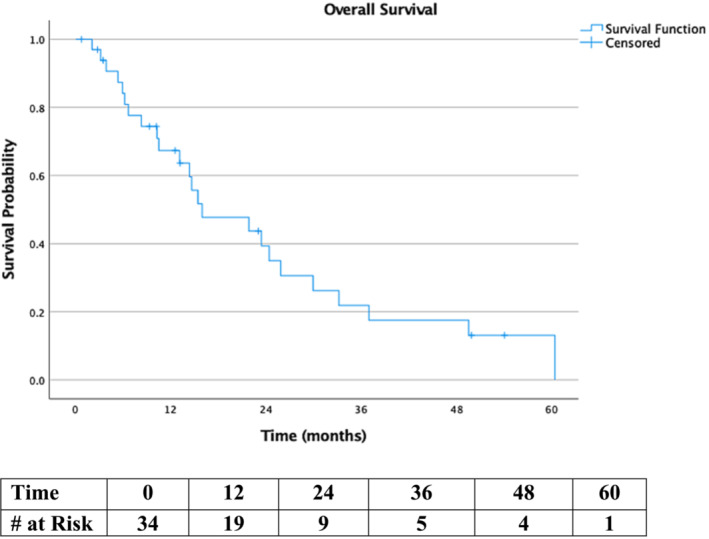
Overall survival by time in months.

Systemic therapy was the mainstay of therapy (chemotherapy *n* = 12; immunotherapy *n* = 5; hormones *n* = 3). Local management included either surgery (*N* = 10) or RT (*n* = 8). Twelve‐ and 24‐month Kaplan–Meier overall survival rate was 67% and 35%, respectively (Figure 1). Total penectomy contributed to local control in seven of eight cases, but no survival benefit was conferred (Mann–Whitney *p* = 0.16).

As might be expected given the rarity of the occurrence, no clinical parameter including primary histology, the presence of synchronous or metachronous metastases, additional metastatic sites or priapism showed statistical survival benefit or detriment.

## 4 DISCUSSION

The mechanism of malignant disease spread to penis has been proposed as venous or lymphatic spread,^4^ based on the robust plexus involving pelvic organs and the dorsal penis. As early as 1919, Ewing noted: ‘The mechanism of the circulation will doubtless explain many of these peculiarities, for there is as yet no evidence that any one … organ is more adapted than others’.^5^ Batson, in 1942, described an injection study in cadavers that yielded ‘valveless vessels which carr[y] blood under low pressures, … constantly subject to arrests and reversals in the direction of the flow of blood’. This phenomenon was termed ‘retrograde transport’.^6^ While this may be true in some cases, more recent data suggest this likely represents too simple a mechanism. Work by Fidler et al. subsequently described site specificity of metastasis of different cell lines,^7^ leading most to reconsider the words of Paget in 1889, describing non‐random patterns of metastasis: ‘When a plant goes to seed, its seeds are carried in all directions; but they can only live and grow if they fall on congenial soil’.^8^

On the other hand, local interruption of lymphovascular flow could certainly be responsible for priapism associated with penile metastasis. Early reports, including one by Young^9^ described priapism related to primary penile cancer. Malignant priapism due to metastasis was first described in 1928 by Begg.^10^ Lin et al. provided a modern update of the phenomenon in 2011.^4^ The frequency of priapism as a presenting symptom in that review was quite frequent (20%–50%), although this may be due to selection bias in published reports. In the current work, overall frequency of priapism was 21% (*n* = 7). Both Lin et al.^4^ and Cocci et al.^1^ proposed that malignant priapism contributed to ‘grim’ prognosis. Our data certainly concur: Median survival of such patients was 6.2 months (range 0.9–15.4 m), although not significantly different from patients without priapism (Mann–Whitney *p* = 0.11).

Not unexpectedly, therapy for such lesions are a function of patient performance status and disease elsewhere. While penectomy is an attractive option clinically for the local disease, it is far less prudent in the presence of multiple other metastases or for an asymptomatic patient otherwise doing poorly. Such patient selection contributed to local control in all but one of our total penectomy cases, but a corresponding survival benefit was not observed (Mann–Whitney *p* = 0.16).

Prior to this report, almost 400 cases of metastasis to penis have been reported since 1870.^4^ Even with the centres of excellence represented here, problems arose both with recalling and with collecting cases since penile metastases are unlikely to be a specific parameter in any database, and resources may not exist to manually retrieve them. Using an automated data retrieval system for the ICD9 code of 198.82 (‘Secondary malignant neoplasm of genital organs’) includes 15 categories for both genders. Finally, even if further sub‐coded by male gender, results include the enormous population of patients with metastatic prostate cancer as well. Commentary made by the contributors included many urethral primary lesions and of primary penile melanoma (*n* > 20) or sarcoma (*n* = 2) cofounding search results.

A specific clinical phenomenon not included here were two cases of extramammary Paget's disease following treatment for urothelial carcinoma of the bladder. We did not consider these strictly to be metastases, since these cases are likely due to intraepithelial spread, not hematogenous. Nineteen other similar cases have been reported separately.^11^

## 5 CONCLUSION

Metastasis to penis arises most frequently from pelvic primaries, although there may be a data retrieval bias in these centres for genitourinary primaries rather than other lesions such as lymphoma. Priapism does not appear to correlate with survival in this large, well‐defined series.

## AUTHOR CONTRIBUTIONS

*Conceptualization*: Philippe E. Spiess and Peter A. S. Johnstone. *Methodology*: Peter A. S. Johnstone. *Formal analysis*: Irini Youssef. *Investigation*: Laura Elst, Nick Watkin, Hielke Martijn de Vries, Chris Protzel and Peter A. S. Johnstone. *Resources*: Oscar Brouwer, Chris Protzel, Benjamin Ayres, Maarten Albersen and Peter A. S. Johnstone. *Data curation*: Laura Elst, Nick Watkin, Hielke Martijn de Vries, Chris Protzel and Peter A. S. Johnstone. *Writing—original draft preparation*: Irini Youssef and Peter A. S. Johnstone. *Writing—review and editing*: Irini Youssef, Nick Watkin, Hielke Martijn de Vries, Oscar Brouwer, Chris Protzel, Benjamin Ayres, Maarten Albersen, Philippe E. Spiess and Peter A. S. Johnstone. *Visualization*: Irini Youssef. *Supervision*: Peter A. S. Johnstone. *Project administration*: Peter A. S. Johnstone. All authors have read and agreed to the published version of the manuscript.

## CONFLICT OF INTEREST STATEMENT

The authors declare no conflict of interest.

## ETHICS STATEMENT

The study was conducted in accordance with the Declaration of Helsinki, and approved by the Institutional Review Board of Moffitt Cancer Center (protocol code MC2171, 10 January 2022).
